# Gender differences in post-stroke cognitive outcomes in rehabilitation: insights from MoCA domain index scores

**DOI:** 10.3389/fneur.2026.1837173

**Published:** 2026-08-05

**Authors:** Valeria Habib, Mariacristina Siotto, Marco Germanotta, Carola Cocco, Valeria Cipollini, Dionysia Papadopoulou, Laura Cortellini, Irene Giovanna Aprile

**Affiliations:** IRCSS Fondazione Don Carlo Gnocchi, Firenze, Italy

**Keywords:** Cognitive Domain Index Scores, cognitive impairment, gender, Montreal Cognitive Assessment, rehabilitation, stroke

## Abstract

Recent literature revealed gender differences in both motor and cognitive deficits after stroke. Women appear to be at a greater disadvantage in terms of recovery, probably for biological and social factors. This post-hoc observational study examined gender differences in global cognitive functioning and across specific cognitive domains in subacute ischemic stroke patients undergoing integrated conventional and robotic multidomain rehabilitation. Cognitive performance was measured before and after a six-week rehabilitation treatment through the Montreal Cognitive Assessment (MoCA). To better characterize domain specific cognitive profiles, the Cognitive Domain Index Scores (CDIS) for executive functions, visuospatial skills, language, attention and orientation were derived from the test. The Brunner–Langer test was performed to account for the main effect of time and the interaction effect between time and gender. The Mann–Whitney *U* test was used to compare CDIS scores between men and women at T0 and T1. We analyzed 66 patients affected by an ischemic insult (69.5 years ± 10.9; 33 women). The sample was gender-balanced and homogeneous for demographic and clinical characteristics. At baseline, women demonstrated lower MoCA compared to men (*p* = 0.018). Following rehabilitation, both groups showed comparable improvements (*p* < 0.001); however, the initial gender difference remained unchanged at discharge. Analysis of the CDIS indicated distinct cognitive profiles between men and women. The CDIS derived from the MoCA allows an easy and rapid identification of domain-specific cognitive deficits, thus providing more detailed information than the global MoCA score. This enables individualized cognitive rehabilitation planning, that also takes gender disparities into account.

## 1 Introduction

Globally, stroke ranks as the second leading cause of mortality and the foremost cause of disability in adults worldwide (1–3) involving somatic motor dysfunction and cognitive decline (4, 5). After stroke, physical and cognitive dysfunction not only compromises patients’ recovery and daily functioning but also leads to higher medical expenses and financial stress for caregivers and society.

Post-stroke cognitive impairment (PSCI), defined as a stroke-induced deficit in one or more cognitive domains (6), is a common consequence of cerebrovascular events, affecting not only older adults but also younger individuals and patients without residual motor deficits (7). Prevalence estimates vary widely, ranging from 7 to 75% (8, 9), depending on factors such as stroke subtype, timing of assessment, pre-stroke cognitive status, number of affected domains, and type of cognitive evaluation used.

Several studies have identified female gender as an independent predictor of post-stroke cognitive decline (10–12).

This may be attributed to both genetic and biological factors and sociocultural factors, such as socioeconomic status and educational level (13). For terminological accuracy, it is important to specify that the term “sex” refers to a set of biological attributes associated with physical, genetic, and physiological characteristics: chromosomes, gene expression, hormonal function, and reproductive/sexual anatomy. Sex is typically classified as female or male, although there are variations in the biological attributes that constitute it and in the way these attributes manifest (14). “Gender” refers to the social roles and behaviors that influence how people perceive themselves and others, how they behave and interact, as well as the distribution of power and resources in society. Although gender is often mistakenly portrayed as binary (male/female), there is in fact a spectrum of gender identities and expressions that define how individuals identify and express their gender (14–16).

In this regard, with respect to sex differences, endocrine variables, notably estrogens, are hypothesized to confer neurovascular protection in women throughout their reproductive years. The abrupt hormonal changes associated with menopause, especially the drop in estrogen levels, may contribute to a sudden increase in stroke risk. The decline in estrogen during menopause causes a corresponding decline in the expression of brain-derived neurotrophic factor (BDNF), a key molecule involved in synaptic plasticity, learning, and memory. This contributes to a reduction in neurotrophic support in women, increasing their vulnerability to ischemic lesions and compromising synaptic plasticity and hinder cognitive recovery processes after a stroke (17–20). Moreover, this may account for the observation that women tend to experience strokes at an older age compared to men (21–23) and are more likely to present with multiple comorbidities (e.g., hypertension, atrial fibrillation) and more severe strokes (24). With regard to gender differences, the literature shows us that women result more prone to social isolation. In fact, women with stroke tend to be more frequently widow, and in some countries, they were often institutionalized before the event (25–30). Beyond hormonal and social factors, the psychological aspects may play also a significant role in shaping post-stroke cognitive recovery. Women are more prone than men to developing post-stroke depression which is a well-established risk factor for cognitive decline (31, 32).

Gender-related differences in post-stroke cognitive profiles are reported in literature. For instance, women more frequently show impairments in attention, executive function, and language, whereas men present with greater deficits in verbal memory (33). These domain-specific differences could have clinical implications, as they can affect the rehabilitation course.

One of the most widely used cognitive screening tools is the Montreal Cognitive Assessment (MoCA). Although MoCA was originally developed to detect Mild Cognitive Impairment and Alzheimer’s disease, it has also proven to be a valuable tool in the assessment of stroke-related cognitive deficits. Unlike other tools as the Mini Mental State Examination (MMSE), the MoCA provides a more detailed assessment of cognitive functions and demonstrates greater sensitivity in detecting PSCI (34, 35). In fact, it includes tasks aimed at assessing executive functions, attention, and abstraction as well as memory-related tasks. For this reason, it is considered more sensitive in detecting PSCI, where prefrontal deficits tend to predominate over memory-related impairments (8, 36). Furthermore, recent studies have shown that MoCA-based screening for cognitive deficits following stroke is both simple and effective (37, 38) and may also serve as a predictor of functional recovery at 3 months (30, 39). Recently, MoCA was also enhanced developing the Cognitive Domain Index Score (CDIS) allowing to account specific scores for memory, executive function, visuospatial abilities, language, attention, and orientation (40), thus providing more detailed information on domain-specific cognitive profiles than the global MoCA score and facilitating rapid cognitive profiling at the individual patient level. These domains have proven capable of providing useful information regarding the functioning of each cognitive domain in the context of dementia (41–43).

This retrospective study aimed to explore gender differences in domain-specific cognitive profiles in a group of subacute ischemic post-stroke patients. The cognitive performance was assessed with the MoCA and CDIS, examining both baseline differences and recovery trajectories following a rehabilitation treatment.

## 2 Materials and methods

### 2.1 Study design and participants

We retrospectively analyzed a group of ischemic subacute post-stroke patients recruited at “S. Maria della Provvidenza” center, Don Carlo Gnocchi Foundation, located in Rome (Italy). On admission (T0), we recorded demographic, medical history, and clinical data. The 56-point Cumulative Illness Rating Scale (CIRS) was assessed to measure disease burden (44).

We recorded information about the stroke lesion location through the Oxfordshire Community Stroke Project Classification (45). Specifically, patients were divided among Lacunal Stroke (LACS), Partial Anterior Circulation Stroke (PACS), Total Anterior Circulation Stroke (TACS), and Posterior Circulation Stroke (POCS).

Inclusion criteria were: first-ever ischemic stroke, confirmed by computed tomography (CT) or magnetic resonance imaging (MRI); age between 18 and 85 years; stroke onset within the previous 6 months; and sufficient cognitive and language abilities to provide informed consent and follow instructions.

Exclusion criteria included a history of previous stroke, the presence of behavioral or cognitive disorders, or reduced compliance that could interfere with active participation in rehabilitation programs or the ability to understand and sign informed consent. This is a secondary analysis of an observational trial originally focused on the nutritional status of post-stroke patients undergoing rehabilitation, and its relationship with functional and cognitive outcomes.

### 2.2 Cognitive assessment

Cognitive performance was assessed using the Italian version of MoCA, a widely used screening tool for detecting mild cognitive impairment and post-stroke cognitive deficits (35). Version A (46) was administered at T0 and version B at T1 (47). Patients were evaluated at admission (T0) and after a 6-week rehabilitation program (T1). This allowed for the evaluation of cognitive changes over the course of the intervention period.

The MoCA CDIS were calculated as described in the study by Julayanont et al. (40). Each CDIS was constructed by grouping MoCA items according to the cognitive domain they are intended to assess, based on evidence from previous neuropsychological and neuroimaging studies. However, certain items may be included in more than one index, as their execution engages multiple cognitive functions and neural networks simultaneously. For example, the items included in the “Attention” section contribute to the calculation of both the Executive Index Score and the Attention Index Score.

The indices described in that study pertain to the following cognitive domains: memory (Memory Index Score – MIS), executive function (Executive Index Score – EIS), visuospatial abilities (Visuospatial Index Score – VIS), language (Language Index Score – LIS), attention (Attention Index Score – AIS), and orientation (Orientation Index Score – OIS).

Specifically, the EIS was obtained by adding the raw scores from the modified Trail Making Test Part B, clock drawing, digit span (forward and backward), letter A tapping, serial-7 subtraction, letter fluency, and abstraction tasks, with a score ranging from 0 to 13. The VIS was calculated by summing the scores from the cube copy, clock drawing, and naming tasks, with a total score ranging from 0 to 7. The LIS included the raw scores from naming, sentence repetition, and letter fluency, with a score ranging from 0 to 6. The AIS was based on the digit span (forward and backward), letter A tapping, serial-7 subtraction, sentence repetition, and the immediate recall trials, with scores ranging from 0 to 18. Finally, the OIS was derived by adding the points obtained in the orientation section of the MoCA, with a maximum score of 6 (Table 1).

**Table 1:** Formulas for calculating MoCA cognitive domain index scores.

MoCA Cognitive Domain Index Scores	Formula	Range
Executive Index Score (EIS)	Trail Making B + Clock Drawing + Digit Span Forward + Digit Span Backward + Letter A Tapping + Serial 7 Subtraction + Letter Fluency + Abstraction	0–13
Visuospatial Index Score (VIS)	Cube Copy + Clock Drawing + Naming	0–7
Language Index Score (LIS)	Naming + Sentence Repetition + Letter Fluency	0–6
Attention Index Score (AIS)	Digit Span Forward + Digit Span Backward + Letter A Tapping + Serial 7 Subtraction + Sentence Repetition + Immediate Recall (Trial 1 + Trial 2)	0–18
Orientation Index Score (OIS)	Orientation Total Score	0–6

**Figure 1:**
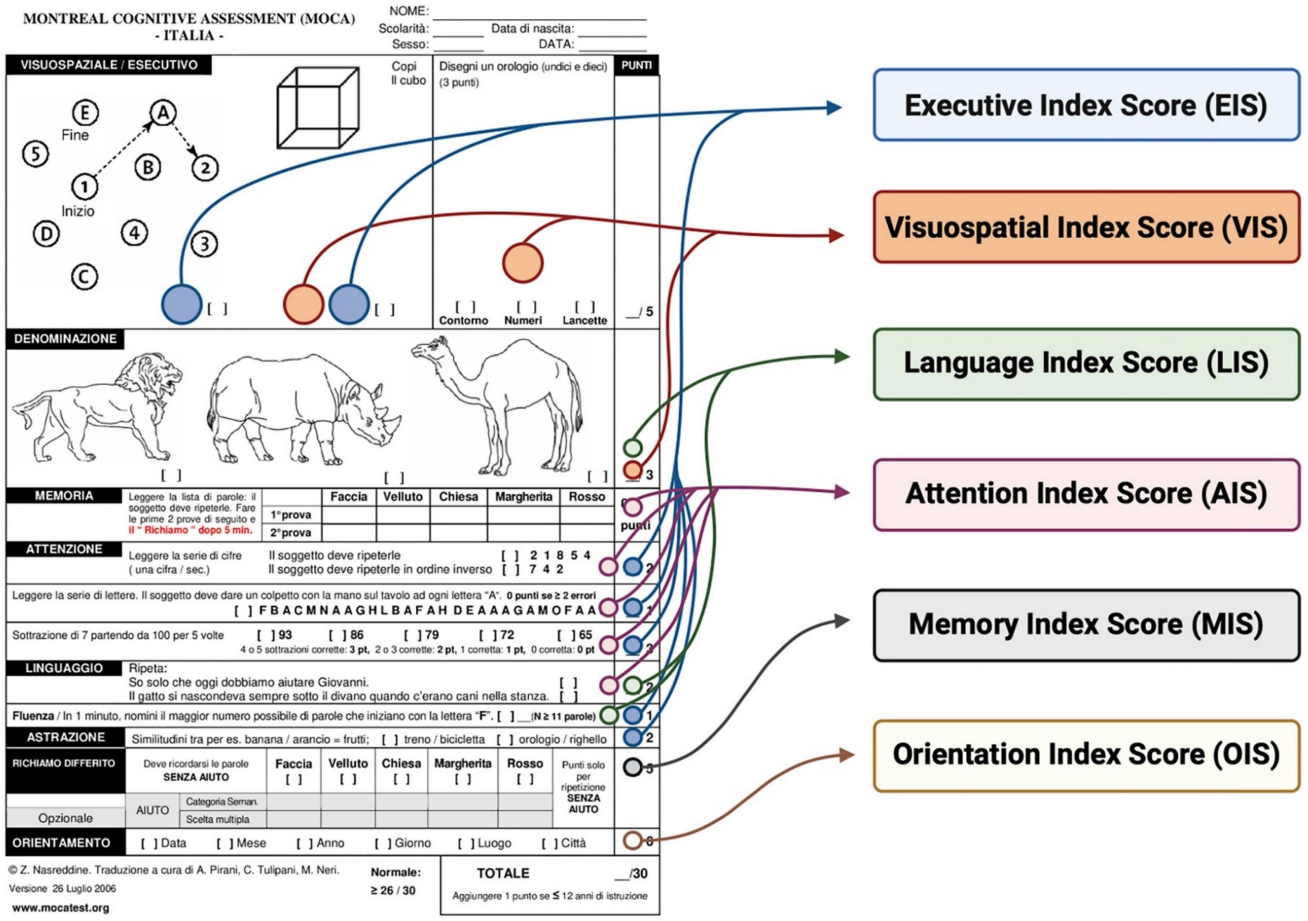
Graphical representation of the items of the Montreal Cognitive Assessment (MoCA) contributing to the Cognitive Domain Index Scores (CDIS). Graphic reworking of the original MoCA test.

The MIS was not calculated, as it requires optional scores of the MoCA that were not included in the original assessment protocol (Figure 1).

### 2.3 Rehabilitation treatment

Patients participated in a six-week rehabilitation program consisting of both conventional physical therapy and robotic therapy. The conventional therapy included sessions lasting 45 min per day, 6 days a week with a combination of active, active-assisted, and passive mobilizations; sensorimotor stimulation; proprioceptive training; motor coordination and balance exercises; postural transitions and transfers; and gait, sitting, and standing training. In addition, patients performed task-oriented activities, such as reaching and grasping objects (e.g., a glass) and practicing activities of daily living (e.g., transfers, dressing, and grooming), tailored to individual functional ability, with the aim of enhancing neuroplasticity and promoting upper limb motor recovery.

Upper limb robotic therapy was provided five times per week for 45 min, employing four technological devices: (i) Amadeo (Tyromotion, Graz, Austria), enabling passive, active, and active-assisted finger flexion and extension of the hand; (ii) Pablo (Tyromotion, Graz, Austria), a sensor-based system allowing for unsupported, three-dimensional movements of the shoulder, elbow, and wrist, both unilaterally and bilaterally; (iii) Diego (Tyromotion, Graz, Austria), a shoulder–arm robotic device supporting unimanual and bimanual shoulder movements in three-dimensional space with weight assistance; and (iv) Motore (Humanware, Pisa, Italy), a robotic system that supports planar shoulder and elbow movements in passive, active, and active-assisted modes (48), (v) Myro (Tyromotion, Graz, Austria), a technological device for manual training of the arm during cognitive task, (vi) Hunova (Movendo Technology, Genoa, Italy) for static and dynamic balance training in sitting and in standing, and (vii) G-EO System (Reha Technology, Olten, Switzerland), an end-effector robot supporting gait training. During robotic training, patients performed both motor and cognitive tasks while receiving real-time visual and auditory feedback. The training of the cognitive component focused on attention, processing speed, executive functions, memory, and visuospatial skills through interactive, game-based activities displayed on a screen integrated into robotic devices. The exercises were organized according to the main cognitive domains targeted, as follows:

- Attention and visual scanning were trained through tasks such as “Trajectories,” “Coins,” “Apple Hunter,” and “Get Green” which required patients to identify target stimuli, track moving objects, and respond selectively while ignoring distractors.
- Memory was stimulated through exercises such as “Memory,” “Grid,” and “Dishwashing,” which involved matching, association, recall, and the execution of predefined action sequences.
- Executive functions were addressed through planning and problem-solving activities, including “Elevator,” “Missing Symbols,” “Road Construction,” and “Hang Up the Laundry,” which required performing sequential actions to achieve a predefined goal.
- Visuospatial skills were trained through navigation, spatial orientation, and construction tasks, such as “Draw by Numbers,” “Grid,” “Road Construction,” and “Crab.”
- Calculation and language-related skills were trained through additional exercises, including “Math Mental” and “Words.”

All participants followed the same cognitive training program regardless of their baseline cognitive profile, while the difficulty of the tasks was progressively adjusted based on individual performance and the severity of motor impairment (49).

All patients underwent speech therapy sessions focused on dysphagia and dysarthria three times a week. Any expressive or linguistic deficits presented by the patients recruited did not affect the comprehension and production necessary for the correct execution of the MoCA.

Before and after rehabilitation treatment, the modified Barthel Index was recorded in all patients to evaluate their ability in daily living activities.

### 2.4 Statistical analysis

Descriptive statistics were reported as means and standard deviations for clinical and demographic variables. Data were tested for normality with Shapiro normality test. Since most variables were not normally distributed, non-parametric tests were used.

Baseline comparisons between men and women were conducted using the non-parametric Mann–Whitney *U* test or the Chi-square test, as appropriate, to determine whether there were any differences in the patients’ clinical and demographic characteristics.

Comparisons of total MoCA score at T0 and CDIS scores at T0 and T1 were performed using the Mann–Whitney U test. Effect sizes for Mann–Whitney *U* tests were estimated using the rank-biserial correlation, with values interpreted as small, moderate, or large effects according to conventional thresholds. To account for multiple comparisons across the five CDIS domains, *p*-values from the Mann–Whitney *U* tests were additionally adjusted using the Benjamini–Hochberg false discovery rate (FDR) procedure. Moreover, to assess whether gender differences were independent of potential confounding variables, additional rank-based ANCOVA models were performed. Cognitive outcome scores were rank-transformed using average ranks for tied values and entered as dependent variables. Gender was included as the independent variable of interest, while age and years of education were included as covariates. A second, fully adjusted model was also performed by additionally including CIRS severity, CIRS comorbidity, and modified Barthel Index at T0 as covariates.

Finally, to assess whether potential changes over time differed significantly between male and female patients, we applied the non-parametric Brunner–Langer test for longitudinal data, to account for both the main effect of time (within-subject factor) and the interaction effect between time and gender (between-subject factor).

A significance threshold of *p* < 0.05 was adopted for all analyses. Statistical analyses were performed using *SPSS* Statistics for Windows, version 29.0.1.0 (SPSS Inc., Chicago, IL, United States) and RStudio version 2026.01.1 (Posit Software, PBC, Boston, MA, United States).

### 2.5 Ethics approval and informed consent

The protocol was approved by the Ethical Committee Comitato Etico Territoriale Lazio Area 1 on 14/05/2025 (prot. 0450/2025). All participants provided written informed consent.

## 3 Results

We analyzed data from 66 subacute post-stroke patients: 33 women and 33 were men. The mean age of the total sample was 69.5 years (SD = 10.9), with a mean age of 71.6 years (SD = 8.7) for women and 67.2 (SD = 12.5) years for men. Table 2 presents the baseline characteristics in terms of age, years of education, time since stroke onset, CIRS Severity Index, CIRS Comorbidity Index, mBI at T0, percentages of patients assuming antidepressants and benzodiazepines at T1. The average time elapsed between the cerebrovascular event and study enrollment was 90.6 days. No statistically significant gender differences were observed for these clinical variables.

**Table 2:** Baseline characteristics of the total sample, and stratified by gender.

Baseline characteristics	Whole group (*n* = 66)	Women (*n* = 33)	Men (*n* = 33)	*p*-value
Age (years)	69.5 ± 10.9	71.6 ± 8.7	67.2 ± 12.5	0.24
Education (years)	10.7 ± 4.2	10.6 ± 4.6	10.8 ± 3.8	0.46
Time since stroke (days)	90.6 ± 39.3	92.6 ± 36.8	88.6 ± 42	0.56
CIRS Severity	2.3 ± 0.4	2.3 ± 0.4	2.2 ± 0.3	0.18
CIRS Comorbidity	5.8 ± 1.7	5.9 ± 1.7	5.6 ± 1.6	0.4
Modified Barthel Index	46.4 ± 19.9	41.3 ± 15.8	51.5 ± 22.4	0.11
Antidepressants T1, *n* (%)	39.4%	42.4%	36.4%	0.61
Benzodiazepines T1, *n* (%)	30.3%	30.3%	30.3%	1.00

Data were reported as mean ± standard deviation or number (%). *p*-values refer to Mann–Whitney *U* test or the chi-squared test, as appropriate.

Similarly, as reported in Table 3, no differences by gender were found in the distribution of lesion site among patients (Table 3). For the purpose of completeness, we declare that we do not have this data for 5 patients (3 women and 2 men).

**Table 3:** Baseline (T0) analysis of lesion sites in 66 subjects of the ischemic subjects divided for Oxfordshire Community Stroke Project Classification (45).

Oxfordshire community stroke project classification	Whole ischemic group (*n* = 66)	Women (*n* = 33)	Men (*n* = 33)
Lacunar Stroke (LACS) *n* (%)	17 (27.9%)	8 (25.8%)	9 (30%)
Partial Anterior Circulation Stroke (PACS) *n* (%)	21 (31.8%)	13 (41.96%)	8 (26.67)
Total Anterior Circulation Stroke (TACS) *n* (%)	13 (19.7%)	8 (25.8%)	5 (16.67%)
Posterior Circulation stroke (POCS) *n* (%)	10 (15.2%)	2 (6.45%)	8 (26.67%)

The data are reported as numbers (%). The Chi-squared test revealed no significant differences in distribution (*χ*^2^ = 5.527; *p* = 0.137).

**Figure 2:**
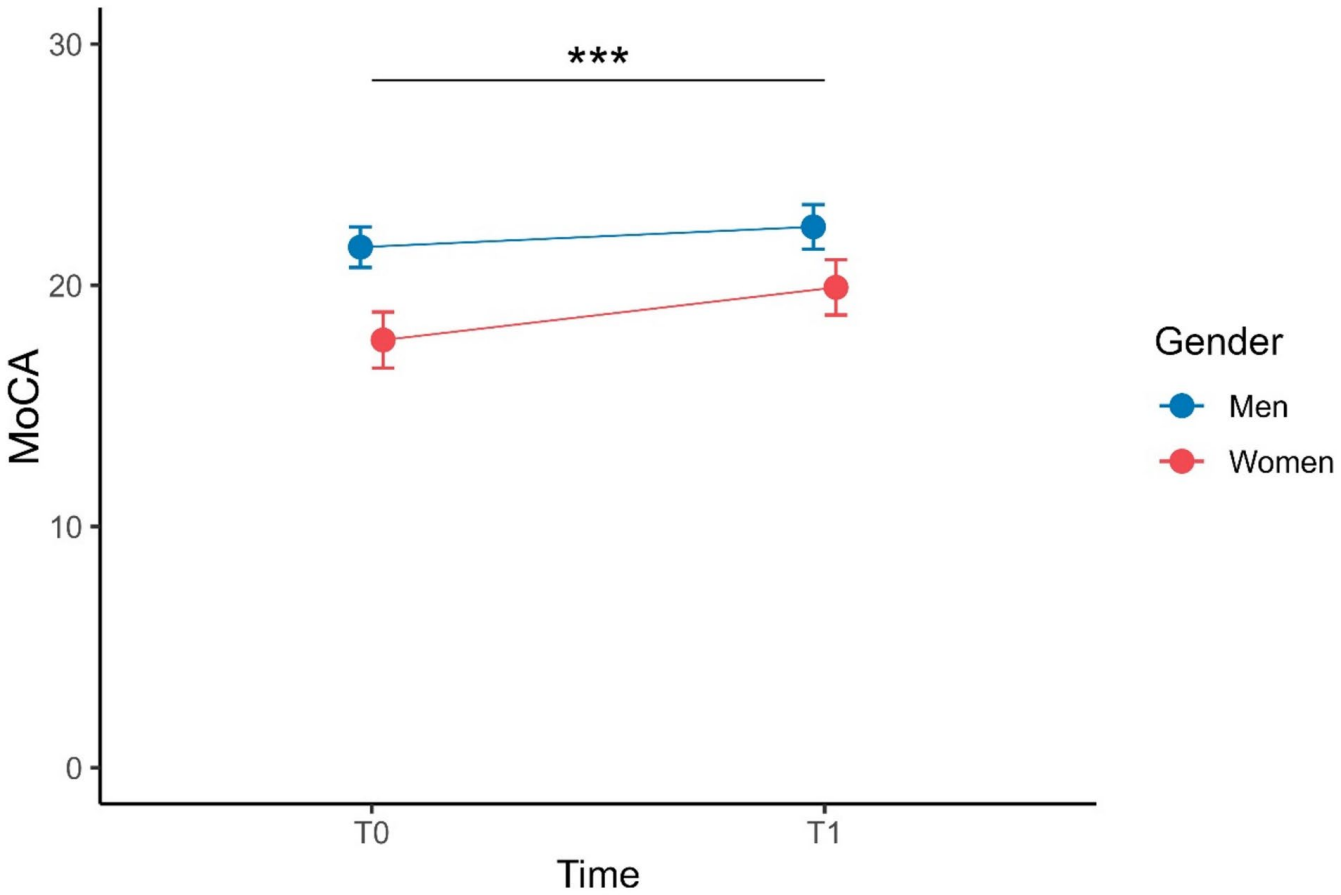
Mean scores with standard errors for the MoCA score measured before (T0) and after (T1) the rehabilitation intervention in male and female participants. Statistical significance of the time effect assessed by the Brunner–Langer nonparametric test is indicated with asterisks (***p < 0.001).

At baseline, the Mann–Whitney *U* test showed a statistically significant gender difference in terms of the total MoCA score, with women’s scores being significantly lower (Men mean = 21.58, SD = 4.84; Women mean = 17.73, SD = 6.67; *p* = 0.018). The Brunner–Langer test revealed a significant main effect of time (*p* < 0.001), indicating that both men and women showed significant improvement in their MoCA total score from T0 to T1. No significant interaction between time and gender emerged, suggesting that cognitive improvement during the rehabilitation period is comparable between men and women (*p* = 0.225) (Figure 2).

The analysis of the MoCA CDIS revealed significant gender differences at baseline in executive functions and visuospatial abilities, with women showing lower performance, as reflected by the EIS and VIS indices (Table 4). These differences were associated with moderate effect sizes (rank-biserial correlation, *r* = 0.308 and *r* = 0.426, respectively). After Benjamini–Hochberg correction for multiple comparisons, the difference in the visuospatial domain remained statistically significant, whereas the executive domain no longer reached statistical significance (adjusted *p* = 0.080). The additional rank-based ANCOVA models confirmed this pattern: after adjustment for age and education, and after further adjustment for CIRS severity, CIRS comorbidity, and mBI, the gender effect remained significant only for the visuospatial domain. These results are reported in Supplementary materials.

**Table 4:** Comparison of cognitive performance measured by the Montreal Cognitive Assessment Score (MoCA) Cognitive Index Domain Scores (CDIS) between women and men at T0.

Cognitive Domain Index Score	Women (*n* = 33)	Men (*n* = 33)	*p*-value	Effect size (*r*)
Executive Index Score (EIS)	6.82 ± 4.00	8.94 ± 3.33	**0.032**	0.308
Visuospatial Index Score (VIS)	4.21 ± 1.83	5.55 ± 1.42	**0.002**	0.426
Language Index Score (LIS)	3.73 ± 1.91	4.58 ± 1.44	0.087	0.241
Attention Index Score (AIS)	11.94 ± 5.14	14.00 ± 4.43	0.075	0.253
Orientation Index Score (OIS)	4.73 ± 1.63	5.06 ± 1.09	0.714	0.051

Data were reported as mean ± standard deviation. *P*-value referred to the Mann–Whitney *U* test. Effect size is reported as rank-biserial correlation (*r*). Values in bold indicate a statistically significant difference (*p* < 0.05). To account for multiple comparisons across CDIS domains, *p*-values were adjusted using the Benjamini–Hochberg false discovery rate (FDR) procedure.

**Figure 3:**
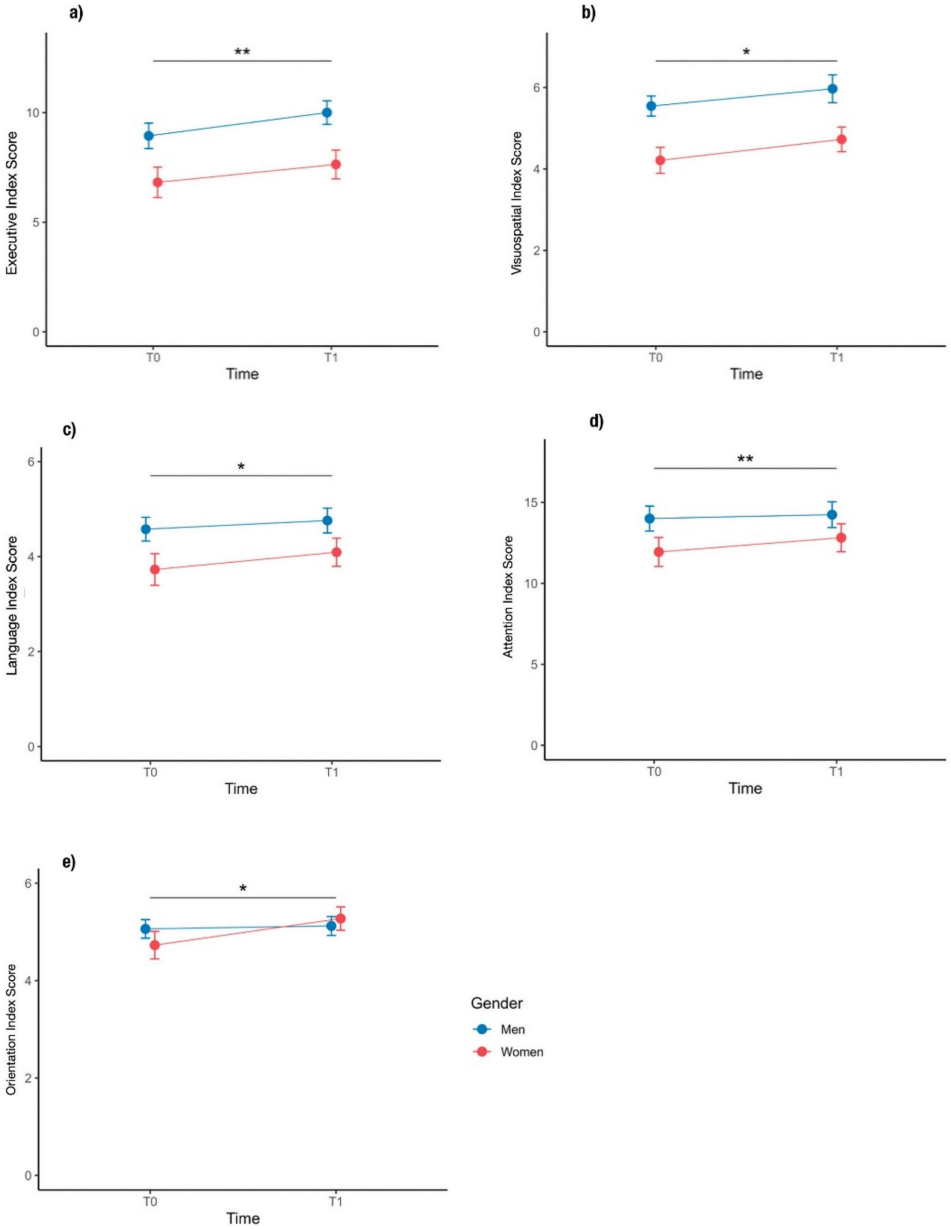
Mean scores with standard errors for the Executive Index Score (EIS) **(a)**, Visuospatial Index Score (VIS) **(b)**, Language Index Score (LIS) **(c)**, Attention Index Score (AIS) **(d)**, Orientation Index Score (OIS) **(e)** measured before (T0) and after (T1) the rehabilitation intervention in male and female participants. Statistical significance of the time effect assessed by the Brunner–Langer nonparametric test is indicated with asterisks (*p < 0.05, **p < 0.01).

The Brunner–Langer test revealed no significant interaction between time and gender for the CDIS, indicating that men and women followed a similar trajectory of cognitive improvement (AIS: *p* = 0.718; EIS: *p* = 0.367; LIS: *p* = 0.918; OIS: *p* = 0.150; VIS: *p* = 0.791). The analysis of main effects indicated that time had a statistically significant effect on all considered variables (MoCA: *p* < 0.001; AIS: *p* = 0.007; EIS: *p* = 0.001; LIS: *p* = 0.023; OIS: *p* = 0.018; VIS: *p* = 0.017) (Figure 3), reflecting an overall improvement after rehabilitation. Furthermore, gender showed a statistically significant main effect on the total MoCA score (*p* = 0.036), EIS (*p* = 0.008), and VIS (*p* = 0.001).

After rehabilitation (T1), EIS and VIS indices were found to be different for gender, consistently with data at admission (Table 5). These differences were associated with moderate effect sizes (rank-biserial correlation, *r* = 0.382 for EIS and *r* = 0.378 for VIS). Both findings remained statistically significant after Benjamini–Hochberg correction for multiple comparisons (adjusted *p* = 0.0175 for both domains) and confirmed by rank-based ANCOVA models (Supplementary materials).

**Table 5:** Comparison of cognitive performance measured by Montreal Cognitive Assessment Score (MoCA) Cognitive Index Domain Scores (CDIS) between women and men at T1.

Cognitive Domain Index Score	Women (*n* = 33)	Men (n = 33)	*p*-value	Effect size (*r*)
Executive Index Score (EIS)	7.64 ± 3.77	10.00 ± 3.09	**0.007**	0.382
Visuospatial Index Score (VIS)	4.73 ± 1.74	5.97 ± 1.96	**0.007**	0.378
Language Index Score (LIS)	4.09 ± 1.70	4.76 ± 1.50	0.080	0.244
Attention Index Score (AIS)	12.82 ± 4.96	14.24 ± 4.57	0.121	0.220
Orientation Index Score (OIS)	5.27 ± 1.38	5.12 ± 1.11	0.271	−0.140

Data were reported as mean ± standard deviation. *P*-values refer to non-parametric Mann–Whitney *U* test. Effect size is reported as rank-biserial correlation (*r*). Values in bold indicate a statistically significant difference (*p* < 0.05). To account for multiple comparisons across CDIS domains, *p*-values were adjusted using the Benjamini–Hochberg false discovery rate (FDR) procedure.

For descriptive purposes, the mean change scores (T1 − T0) for each index of the cognitive domain (CDIS) of the MoCA are reported separately for women and men (Table 6), providing an estimate of the extent of cognitive improvement in each group.

**Table 6:** Mean values of the changes (T1 − T0) for each cognitive domain index (CDIS) of the Montreal Cognitive Assessment (MoCA) in women and men.

Cognitive Domain Index Score	Main effect (Women = 33)	Main effect (Men = 33)
Executive Index Score (EIS)	0.31 ± 1.03	0.4 ± 1.64
Visuospatial Index Score (VIS)	0.2 ± 0.85	0.16 ± 1.24
Language Index Score (LIS)	0.14 ± 0.84	0.07 ± 0.37
Attention Index Score (AIS)	0.33 ± 1.3	0.09 ± 1.39
Orientation Index Score (OIS)	0.2 ± 0.84	0.02 ± 0.48

The data are presented as mean ± standard deviation.

## 4 Discussion

In this study, we analyze gender differences in post-stroke cognitive outcomes through the MoCA and the Cognitive Domain Index Scores (CDIS), in a sample of 66 subacute ischemic patients who underwent a six-week of integrated conventional and robotic rehabilitation.

The study revealed three main findings:

(1) All patients analyzed responded positively to treatment, achieving a significant increase in MoCA scores at the end of treatment;

(2) At the start of the study, women had significantly lower cognitive levels than men. Although they showed significant improvement, their performance still remained lower compared to men;

(3) CDIS provided a more detailed characterization of the cognitive profile, making it possible to identify gender differences. In particular, it emerged that women are more affected than men in terms of executive and visuospatial abilities.

To our knowledge, this is the first time that these indices have been tested in post-stroke subjects undergoing rehabilitation.

We found a significant improvement in cognitive performance across the entire sample following the rehabilitation treatment. This result may be associated with the rehabilitation program, which integrated conventional rehabilitation and robotic training. This interpretation is consistent with previous findings by Aprile et al. (49), who reported significant post-intervention improvements in several cognitive domains, such as attention, executive functioning, memory, and visuospatial skills, following a structured robotic upper limb training program in post-stroke patients. The authors emphasized the role of combining motor and cognitive stimulation in a single rehabilitation setting, suggesting that robotics-based interventions can improve not only motor, but also cognitive recovery through targeted and engaging exercises. Their findings support the idea that robotic rehabilitation can facilitate domain-general cognitive gains, as detected by domain-specific tools, such as: Oxford Cognitive Screen (OCS), Symbol Digit Modalities Test, Digit Span, Rey–Osterrieth Complex Fig., Tower of London, Stroop Color and Word Test (49). Nevertheless, the present study design does not allow the specific contribution of robotic intervention to be disentangled from spontaneous recovery, conventional rehabilitation, or other nonspecific effects.

Another aspect that emerged from our analyses concerns the trajectories of cognitive change following the rehabilitation treatment. Both men and women benefit equally from rehabilitation treatment, exhibiting a comparable response. However, regardless of the assessment time, women show lower overall cognitive performance than the men. This result is consistent with literature published on this gender gap found in post-stroke cognitive outcomes (10–12). As suggested the women disadvantage can depend on social (25–30), clinical (more comorbidities) (24), physiological (estrogen protective factor failure or decrease after menopause) (17–20), psychological (greater propensity to develop post-stroke depression) variables (31, 32).

Domain-specific analysis using the CDIS enabled the identification of gender-related differences in cognitive profiles that otherwise could not be identified by examining the overall MoCA score. This result is particularly significant given the evidence that there is no statistically significant gender difference in the location of ischemic lesions. Before rehabilitation, women exhibited significantly lower performance in executive functioning and visuospatial abilities compared to men. Following treatment – although both groups showed improvement in general cognitive performance – these gender disparities persisted. This highlights the fact that while women showed both general and domain specific cognitive improvement after robotic rehabilitation, some higher-order cognitive functions remain more impaired when compared with men. Such a pattern is consistent with previous studies that found a gender-related vulnerability in executive and attention after stroke. A multicenter study in 2,343 patients with acute ischemic stroke revealed that, while the overall prevalence of post-stroke cognitive impairment (PSCI) was comparable between men and women (51% in both men and women), the affected cognitive domains differed significantly. Women were more likely to exhibit impairments in attention and executive functioning as well as in language abilities, whereas men showed a higher prevalence of deficits in verbal memory. Moreover, the multicenter study reported that these gender-specific patterns persisted even after adjusting for age and education level. Such differences in cognitive profiles suggest that men and women may experience distinct cognitive challenges after stroke, which should be considered in both clinical assessments and individualized rehabilitation strategies (33).

The identification of lower executive and visuospatial performance in women may have potential clinical implications. CDIS may provide a preliminary characterization of domain-specific cognitive vulnerabilities and help identify patients who could benefit from further neuropsychological assessment. In particular, the lower executive and visuospatial performance observed in women may indicate the need for a more in-depth evaluation of these domains. When such deficits are confirmed through comprehensive assessment, evidence-based interventions targeting executive functions (e.g., metacognitive strategy training and problem-solving approaches) (50) and visuospatial abilities (e.g., visual scanning and spatial exploration training) may be considered (51). However, the present study did not evaluate the efficacy of such targeted interventions, and therefore these implications should be considered preliminary. A key strength of this method is its reliance on a brief and easily administered tool, capable of providing clinicians with an initial overview of cognitive functioning and guiding further diagnostic and therapeutic planning. Clearly, there are other assessment tools that allow for a detailed evaluation of various cognitive domains, but this takes time. It is not always possible in all clinical settings to devote so much time to the clinical and cognitive evaluation of a patient.

This study has some limitations inherent in its retrospective design. First of all, only data from one of the centers originally involved in the multicenter study were included in this exploratory analysis.

Secondly, the relatively small sample size may have limited the statistical power needed to detect subtle gender differences in specific cognitive domains, particularly given the multiple CDIS comparisons conducted. Therefore, non-significant results should be interpreted with caution, as they may reflect limited statistical power rather than the absence of actual differences. Although significant differences between groups were associated with moderate effect sizes, some non-significant comparisons showed small to moderate effect sizes, suggesting that further differences may emerge in larger cohorts. Future studies involving larger, multicenter samples with adequate statistical power are needed to confirm and expand upon these findings.

As mentioned, the data were originally collected for a different primary purpose, and the present analysis was conducted retrospectively to explore cognitive aspects that were not predefined in the initial protocol. As a result, some variables necessary for calculating specific MoCA cognitive index scores, such as the MIS were not available, as the corresponding optional items had not been systematically recorded during the original data collection. This limitation prevented a comprehensive characterization of domain-specific cognitive profiles. Given that memory impairment is a common consequence of stroke and that previous studies have reported potential sex-related differences in mnemonic performance, the absence of the MIS may have limited our ability to identify specific memory-related differences between men and women. Consequently, the cognitive models identified using the CDIS should be considered a partial rather than a comprehensive representation of post-stroke cognitive functioning.

Another limitation concerns the characterization of the cognitive component of the rehabilitation intervention. Although all participants received the same standardized motor-cognitive training protocol, no specific measures of cognitive workload, task performance, or cognitive engagement were collected during treatment. Therefore, it was not possible to quantify the intensity of cognitive stimulation received by each participant or to examine its relationship with cognitive outcomes.

A further consideration is the use of Cognitive Domain Index (CDIS) scores, derived from the MoCA, which is a screening tool rather than a comprehensive neuropsychological battery. Although the CDIS scores have provided a more detailed characterization of cognitive profiles than the MoCA total score alone, their application in stroke population remains relatively unexplored. To date, CDIS scores have been studied primarily in populations with dementia and mild cognitive impairment, and further studies are needed to establish their validity, reliability, and clinical utility in post-stroke cognitive assessment and rehabilitation settings.

Finally, as a retrospective study, it did not include a comparison between MoCA CDIS and other domain-specific cognitive measures, which would have allowed a more accurate interpretation of the cognitive profiles.

Further studies involving larger and multicenter samples are needed to replicate these findings and to better clarify the sensitivity of the MoCA CDIS in the subacute rehabilitation phase. In this regard we are conducting two multicenters ambitious studies (StrokeFit4 NCT06547827 and the Fit4MedRobStroke@Home; NCT06978413) in the Fit4 Medical Robotics project (FIT4MEDROB), that will permit to deepen into the role and efficacy of robotic rehabilitation and telerehabilitation also in relation to cognitive performances measured with MoCA.

## 5 Conclusion

The present study investigated gender differences in post-stroke cognitive performance within a rehabilitation setting, in which robotic programs are integrated with conventional ones. The results show significant cognitive improvement in both men and women, consistent with the expected efficacy of the intervention. However, in line with findings from part of the existing literature, women exhibited greater cognitive impairment than men both at the beginning and at the end of the rehabilitation treatment, despite overall cognitive improvement. The use of the CDIS of MoCA enables more detailed characterization of the cognitive profiles of the two groups, highlighting its clinical utility as a quick and effective tool for identifying post-stroke cognitive profiles.

## Data Availability

The datasets generated and analyzed during the current study are not publicly available due to institutional policy and ethical considerations related to patient data but are available from the corresponding author on reasonable request. Requests to access the datasets should be directed to msiotto@dongnocchi.it.
