# Supplementary material for: Gender differences in post-stroke cognitive outcomes in rehabilitation: insights from MoCA domain index scores
Source: Front Neurol. 2026 Aug 5;17:1837173. doi: 10.3389/fneur.2026.1837173 (PMC13487415; doi:10.3389/fneur.2026.1837173)
Supplement: Supplementary file 1 [file Data_Sheet_1.PDF]

Supplementary Materials

Rank-based ANCOVA models

Rank-based ANCOVA models were performed using rank-transformed cognitive outcomes as dependent variables, in order to evaluate whether the observed gender-related differences were independent of potential confounding variables. This approach was selected to maintain a non-parametric analytical framework while allowing adjustment for relevant covariates.

The cognitive outcomes included the Montreal Cognitive Assessment (MoCA) Cognitive Domain Index Scores (CDIS), namely the Executive Index Score (EIS), Language Index Score (LIS), Visuospatial Index Score (VIS), Attention Index Score (AIS), and Orientation Index Score (OIS), assessed at baseline (T0) and follow-up (T1).

Two models were fitted for each cognitive outcome. Model 1 included gender as the independent variable of interest, with age and years of education entered as covariates, and was specified as follows:

- Model 1: rank(cognitive outcome) ~ gender + age + education.

Model 2 was designed as a fully adjusted model and additionally accounted for comorbidity burden and functional status. Specifically, Cumulative Illness Rating Scale (CIRS) severity, CIRS comorbidity, and modified Barthel Index (mBI) were added as covariates. Model 2 was specified as follows:

- Model 2: rank(cognitive outcome) ~ gender + age + education + CIRS severity + CIRS comorbidity + mBI.

Gender was coded as 0 = men and 1 = women. For each model, regression estimates, standard errors, test statistics, p-values, 95% confidence intervals, and overall model fit indices are reported. Bold values and bold variable names indicate statistically significant effects ($p < 0.05$).

Dependent Variable: MOCA Executive Index Score (EIS) at baseline (T0)

Model 1

term	estimate	std.error	statistic	p.value	conf.low
gender	-8.228	4.233	-1.944	0.056	-16.689 to 0.232
age	-0.328	0.198	-1.659	0.102	-0.724 to 0.067
**education**	1.984	0.504	3.936	**<0.001**	0.976 to 2.991

Model fit: n = 66; R^2 = 0.266; adjusted R^2 = 0.231; F(3, 62) = 7.496; model p = <0.001

Model 2

term	estimate	std.error	statistic	p.value	conf.low
gender	-6.632	4.342	-1.528	0.132	-15.320 to 2.056
age	-0.315	0.213	-1.482	0.144	-0.741 to 0.110
**education**	1.913	0.515	3.717	**<0.001**	0.883 to 2.942
CIRS severity	-7.688	14.743	-0.522	0.604	-37.188 to 21.811
CIRS comorbidity	2.645	3.134	0.844	0.402	-3.626 to 8.917
mBI T0	0.172	0.116	1.489	0.142	-0.059 to 0.403

Model fit: n = 66; R^2 = 0.304; adjusted R^2 = 0.233; F(6, 59) = 4.292; model p = 0.001

Dependent Variable: MOCA Language Index Score (LIS) at baseline (T0)

Model 1

term	estimate	std.error	statistic	p.value	95% CI
gender	-6.653	4.589	-1.450	0.152	-15.826 to 2.519
age	-0.234	0.215	-1.089	0.280	-0.663 to 0.195
education	1.021	0.546	1.869	0.066	-0.071 to 2.113

Model fit: n = 66; R^2 = 0.104; adjusted R^2 = 0.061; F(3, 62) = 2.410; model p = 0.075

Model 2

term	estimate	std.error	statistic	p.value	95% CI
gender	-5.985	4.809	-1.244	0.218	-15.608 to 3.638
age	-0.242	0.236	-1.027	0.308	-0.714 to 0.229
education	1.003	0.570	1.759	0.084	-0.138 to 2.143
CIRS severity	-4.059	16.330	-0.249	0.805	-36.735 to 28.617
CIRS comorbidity	1.521	3.472	0.438	0.663	-5.426 to 8.468
mBI T0	0.070	0.128	0.548	0.586	-0.186 to 0.326

Model fit: n = 66; R^2 = 0.113; adjusted R^2 = 0.023; F(6, 59) = 1.255; model p = 0.292

Dependent Variable: MOCA Visuospatial Index Score (VIS) at baseline (T0)

Model 1

term	estimate	std.error	statistic	p.value	95% CI
**gender**	-12.173	4.269	-2.852	**0.006****	-20.706 to -3.640
age	-0.358	0.200	-1.795	0.077	-0.758 to 0.041
**education**	1.166	0.508	2.295	**0.025**	0.150 to 2.182

Model fit: n = 66; R^2 = 0.231; adjusted R^2 = 0.194; F(3, 62) = 6.218; model p = <0.001

Model 2

term	estimate	std.error	statistic	p.value	95% CI
**gender**	-10.578	4.396	-2.406	**0.019***	-19.374 to -1.782
age	-0.292	0.215	-1.355	0.181	-0.723 to 0.139
**education**	1.106	0.521	2.122	**0.038***	0.063 to 2.148
CIRS severity	-13.744	14.926	-0.921	0.361	-43.611 to 16.124
CIRS comorbidity	2.254	3.173	0.710	0.480	-4.095 to 8.604
mBI T0	0.129	0.117	1.101	0.275	-0.105 to 0.363

Model fit: n = 66; R^2 = 0.265; adjusted R^2 = 0.190; F(6, 59) = 3.547; model p = 0.005

Dependent Variable: MOCA Attention Index Score at baseline (T0)

Model 1

term	estimate	std.error	statistic	p.value	95% CI
gender	-5.867	4.286	-1.369	0.176	-14.435 to 2.700
**age**	-0.462	0.200	-2.307	**0.024***	-0.863 to -0.062
**education**	1.794	0.510	3.516	**<0.001*****	0.774 to 2.814

Model fit: n = 66; R^2 = 0.237; adjusted R^2 = 0.200; $F(3, 62)$ = 6.428; model p = <0.001

Model 2

term	estimate	std.error	statistic	p.value	95% CI
gender	-4.015	4.346	-0.924	0.359	-12.711 to 4.680
age	-0.367	0.213	-1.724	0.090	-0.793 to 0.059
**education**	1.607	0.515	3.121	**0.003****	0.577 to 2.638
CIRS severity	3.010	14.756	0.204	0.839	-26.516 to 32.537
CIRS comorbidity	-0.228	3.137	-0.073	0.942	-6.506 to 6.049
**mBI T0**	0.249	0.116	2.150	**0.036***	0.017 to 0.480

Model fit: n = 66; R^2 = 0.293; adjusted R^2 = 0.221; $F(6, 59)$ = 4.075; model p = 0.002

Dependent Variable: MOCA Orientation Index Score (OIS) at baseline (T0)

Model 1

term	estimate	std.error	statistic	p.value	95% CI
gender	-0.712	4.622	-0.154	0.878	-9.951 to 8.527
age	-0.185	0.216	-0.854	0.397	-0.617 to 0.248
education	0.522	0.550	0.948	0.347	-0.578 to 1.622

Model fit: n = 66; R^2 = 0.024; adjusted R^2 = -0.023; $F(3, 62)$ = 0.507; model p = 0.679

Model 2

term	estimate	std.error	statistic	p.value	95% CI
gender	-1.600	4.775	-0.335	0.739	-11.156 to 7.955
age	-0.131	0.234	-0.561	0.577	-0.600 to 0.337
education	0.533	0.566	0.941	0.351	-0.600 to 1.665
CIRS severity	4.505	16.215	0.278	0.782	-27.941 to 36.951
CIRS comorbidity	-2.829	3.447	-0.821	0.415	-9.727 to 4.069
mBI T0	-0.106	0.127	-0.836	0.406	-0.360 to 0.148

Model fit: n = 66; R^2 = 0.061; adjusted R^2 = -0.035; $F(6, 59)$ = 0.635; model p = 0.701

Dependent Variable: MOCA Executive Index Score (EIS) after rehabilitation (T1)

Model 1

term	estimate	std.error	statistic	p.value	95% CI
**gender**	-11.350	4.198	-2.704	**0.009****	-19.741 to -2.959
age	-0.188	0.196	-0.958	0.342	-0.581 to 0.204
**education**	1.862	0.500	3.726	**<0.001*****	0.863 to 2.861

Model fit: n = 66; R^2 = 0.274; adjusted R^2 = 0.239; F(3, 62) = 7.816; model p = <0.001

Model 2

term	estimate	std.error	statistic	p.value	95% CI
**gender**	-10.612	4.362	-2.433	**0.018***	-19.341 to -1.883
age	-0.217	0.214	-1.015	0.314	-0.645 to 0.211
**education**	1.835	0.517	3.549	**<0.001*****	0.800 to 2.869
CIRS severity	-1.790	14.813	-0.121	0.904	-31.430 to 27.850
CIRS comorbidity	1.761	3.149	0.559	0.578	-4.540 to 8.062
mBI T0	0.096	0.116	0.825	0.412	-0.136 to 0.328

Model fit: n = 66; R^2 = 0.294; adjusted R^2 = 0.222; F(6, 59) = 4.085; model p = 0.002

Dependent Variable: MOCA Language Index Score (LIS) after rehabilitation (T1)

Model 1

term	estimate	std.error	statistic	p.value	95% CI
gender	-6.941	4.403	-1.576	0.120	-15.743 to 1.861
age	-0.174	0.206	-0.845	0.401	-0.586 to 0.238
**education**	1.523	0.524	2.906	**0.005****	0.475 to 2.571

Model fit: n = 66; R^2 = 0.163; adjusted R^2 = 0.123; F(3, 62) = 4.025; model p = 0.011

Model 2

term	estimate	std.error	statistic	p.value	95% CI
gender	-6.097	4.533	-1.345	0.184	-15.167 to 2.974
age	-0.234	0.222	-1.056	0.295	-0.679 to 0.210
**education**	1.517	0.537	2.823	**0.006****	0.442 to 2.592
CIRS severity	-4.131	15.392	-0.268	0.789	-34.931 to 26.669
CIRS comorbidity	2.885	3.272	0.882	0.382	-3.663 to 9.433
mBI T0	0.104	0.121	0.859	0.394	-0.138 to 0.345

Model fit: n = 66; R^2 = 0.200; adjusted R^2 = 0.119; $F(6, 59)$ = 2.464; model p = 0.034

Dependent Variable: MOCA Visuospatial Index Score (VIS) after rehabilitation (T1)

Model 1

term	estimate	std.error	statistic	p.value	95% CI
**gender**	-11.147	4.234	-2.633	**0.011***	-19.611 to -2.683
age	-0.219	0.198	-1.107	0.273	-0.615 to 0.177
**education**	1.580	0.504	3.133	**0.003****	0.572 to 2.587

Model fit: n = 66; R^2 = 0.237; adjusted R^2 = 0.200; F(3, 62) = 6.422; model p = <0.001

Model 2

term	estimate	std.error	statistic	p.value	95% CI
**gender**	-11.213	4.381	-2.560	**0.013***	-19.979 to -2.448
age	-0.317	0.215	-1.478	0.145	-0.747 to 0.112
**education**	1.640	0.519	3.158	**0.003****	0.601 to 2.679
CIRS severity	-1.933	14.875	-0.130	0.897	-31.697 to 27.832
CIRS comorbidity	2.323	3.162	0.735	0.465	-4.005 to 8.651
mBI T0	-0.000	0.117	-0.004	0.997	-0.234 to 0.233

Model fit: n = 66; R^2 = 0.264; adjusted R^2 = 0.189; F(6, 59) = 3.524; model p = 0.005

Dependent Variable: MOCA Attention Index Score (AIS) after rehabilitation (T1)

Model 1

term	estimate	std.error	statistic	p.value	95% CI
gender	-5.778	4.450	-1.299	0.199	-14.672 to 3.117
age	-0.249	0.208	-1.195	0.237	-0.665 to 0.167
**education**	1.684	0.530	3.180	**0.002****	0.625 to 2.743

Model fit: n = 66; R^2 = 0.177; adjusted R^2 = 0.137; F(3, 62) = 4.442; model p = 0.007

Model 2

term	estimate	std.error	statistic	p.value	95% CI
gender	-4.660	4.603	-1.013	0.315	-13.870 to 4.549
age	-0.206	0.226	-0.913	0.365	-0.657 to 0.245
**education**	1.555	0.546	2.850	**0.006****	0.463 to 2.647
CIRS severity	5.910	15.628	0.378	0.707	-25.362 to 37.181
CIRS comorbidity	-0.347	3.322	-0.104	0.917	-6.995 to 6.302
mBI T0	0.175	0.122	1.429	0.158	-0.070 to 0.420

Model fit: n = 66; R^2 = 0.206; adjusted R^2 = 0.125; F(6, 59) = 2.553; model p = 0.029

Dependent Variable: MOCA Orientation Index Score (OIS) after rehabilitation (T1)

Model 1

term	estimate	std.error	statistic	p.value	95% CI
gender	5.794	4.273	1.356	0.180	-2.747 - 14.335
age	-0.224	0.200	-1.123	0.266	-0.624 - 0.175
education	0.620	0.509	1.219	0.227	-0.397 - 1.637

Model fit: n = 66; R^2 = 0.054; adjusted R^2 = 0.009; $F(3, 62)$ = 1.190; model p = 0.321

Model 2

term	estimate	std.error	statistic	p.value	95% CI
gender	6.806	4.454	1.528	0.132	-2.106 to 15.719
age	-0.226	0.218	-1.035	0.305	-0.663 to 0.211
education	0.609	0.528	1.153	0.253	-0.448 to 1.665
CIRS severity	-9.969	15.124	-0.659	0.512	-40.232 to 20.294
CIRS comorbidity	2.554	3.215	0.794	0.430	-3.880 to 8.988
mBI T0	0.083	0.119	0.704	0.485	-0.154 to 0.320

Model fit: n = 66; R^2 = 0.074; adjusted R^2 = -0.021; $F(6, 59)$ = 0.782; model p = 0.588
